# Transfer of the Integrative and Conjugative Element ICE*St3* of Streptococcus thermophilus in Physiological Conditions Mimicking the Human Digestive Ecosystem

**DOI:** 10.1128/spectrum.04667-22

**Published:** 2023-03-30

**Authors:** Pauline Herviou, Aurélie Balvay, Deborah Bellet, Sophie Bobet, Claire Maudet, Johan Staub, Monique Alric, Nathalie Leblond-Bourget, Christine Delorme, Sylvie Rabot, Sylvain Denis, Sophie Payot

**Affiliations:** a Université Clermont-Auvergne, INRAE, MEDIS, Clermont-Ferrand, France; b Université Paris-Saclay, INRAE, AgroParisTech, Micalis Institute, Jouy-en-Josas, France; c Université de Lorraine, INRAE, DynAMic, Nancy, France; University at Albany

**Keywords:** ARCOL, integrative conjugative element, TIM, conjugation, digestive tract, gene transfer, mice, mobile genetic elements

## Abstract

Metagenome analyses of the human microbiome suggest that horizontal gene transfer (HGT) is frequent in these rich and complex microbial communities. However, so far, only a few HGT studies have been conducted *in vivo*. In this work, three different systems mimicking the physiological conditions encountered in the human digestive tract were tested, including (i) the TNO gastro-Intestinal tract Model 1 (TIM-1) system (for the upper part of the intestine), (ii) the ARtificial COLon (ARCOL) system (to mimic the colon), and (iii) a mouse model. To increase the likelihood of transfer by conjugation of the integrative and conjugative element studied in the artificial digestive systems, bacteria were entrapped in alginate, agar, and chitosan beads before being placed in the different gut compartments. The number of transconjugants detected decreased, while the complexity of the ecosystem increased (many clones in TIM-1 but only one clone in ARCOL). No clone was obtained in a natural digestive environment (germfree mouse model). In the human gut, the richness and diversity of the bacterial community would offer more opportunities for HGT events to occur. In addition, several factors (SOS-inducing agents, microbiota-derived factors) that potentially increase *in vivo* HGT efficiency were not tested here. Even if HGT events are rare, expansion of the transconjugant clones can happen if ecological success is fostered by selecting conditions or by events that destabilize the microbial community.

**IMPORTANCE** The human gut microbiota plays a key role in maintaining normal host physiology and health, but its homeostasis is fragile. During their transit in the gastrointestinal tract, bacteria conveyed by food can exchange genes with resident bacteria. New traits acquired by HGT (e.g., new catabolic properties, bacteriocins, antibiotic resistance) can impact the gut microbial composition and metabolic potential. We showed here that TIM-1, a system mimicking the upper digestive tract, is a useful tool to evaluate HGT events in conditions closer to the physiological ones. Another important fact pointed out in this work is that Enterococcus faecalis is a good candidate for foreign gene acquisition. Due to its high ability to colonize the gut and acquire mobile genetic elements, this commensal bacterium could serve as an intermediate for HGT in the human gut.

## INTRODUCTION

The human gut microbiota plays a key role in maintaining normal host physiology and health (1, 2). In addition to its role in digestion and metabolism, it participates in the defense against pathogens and the development of host immune response and also influences brain-gut communication (1, 2). An imbalance of the ecosystem (dysbiosis) can lead to the development of diseases (inflammatory bowel diseases, obesity, or colorectal cancer) (1, 2). The microbial community that inhabits the human gut is complex, consisting of more than 1,500 species distributed in more than 50 phyla. However, a few phyla, which include *Firmicutes* and *Bacteroides*, are dominant and constitute up to 90% of the total microbial population (2). The total gut microbiota reaches up to 10^13^ cells, thus being in the same order of magnitude as the number of human cells (3). The gut microbiota varies according to the intestine region due to differences in pH, oxygen content, digestive flow rate, substrate availability, and host secretions. Hence, the large intestine (colon), which is characterized by slower flow rates and less deleterious conditions (e.g., digestive secretions) than the small intestine, offers conditions more propitious to microorganism life and thus hosts a larger microbial community (4). Although gut microbiota is partly determined by host genetics (5), environmental factors, in particular, diet and antibiotic use, largely impact microbiota composition (6–8).

During their transit in the gastrointestinal tract (GIT), bacteria conveyed by food (bacteria used in the food industry, probiotics, or contaminants of food) can exchange genes with resident bacteria (9). New traits acquired by horizontal gene transfer (HGT) can include defense against stresses, new catabolic properties (catabolism of sugars or other nutriments found in the ecosystem), competitive weapons such as bacteriocins, adhesion, and virulence properties, but also antibiotic resistance (10). HGT can thus modify the equilibrium of the intestinal microbiota by giving new competitive advantages to some strains or leading to the emergence of new pathogens or increasing the virulence of existing ones (11, 12). HGT is favored by the high bacterial population in the colon, the multiple opportunities of conjugation offered on the surfaces of food particles and host tissues, and the exposure of bacteria to stresses and antibiotic treatments in the human gut (13, 14). The presence of mucus that prevents efficient mixing of the bacterial cells could also create microniches where efficient and rapid gene transfer can take place (15).

Mobile genetic elements (MGEs) play a key role in HGT in bacteria (16, 17). The multiplication of bacterial genome sequencing projects in the last decade provides a remarkable opportunity to explore the diversity and prevalence of bacterial mobile genetic elements. These analyses shed light on elements integrated into the chromosome called integrative and conjugative elements (ICEs), which are still less known than plasmids despite their high prevalence in bacteria (18). ICEs encode proteins for their own excision, transfer by conjugation, and integration (19). In addition to the genes involved or controlling their mobility, all ICEs carry cargo genes, which can provide properties (adaptation, virulence, antibiotic resistance) advantageous for the bacterial host (19, 20). Numerous ICEs have been identified in the genomes of streptococci (21, 22), bacteria that belong to the *Firmicutes* phylum, one of the dominant bacterial groups in the human intestinal microbiota. Many of them belong to the ICE*St3-*Tn*916* superfamily, known to carry antibiotic-resistance genes (21, 23). One member of this superfamily, ICE*St3*, first described in Streptococcus thermophilus (24), a bacterium used in the dairy industry, was shown to transfer efficiently in the S. thermophilus species (around 10^−4^ transconjugant per donor cell) as well as to Enterococcus faecalis (25). Related ICEs were also detected in many other streptococci (in particular, Streptococcus salivarius) that are natural inhabitants or transit in the human GIT (22, 26–28).

Studies on HGT were mostly done *in vitro*, and there is still a significant knowledge gap regarding *in situ* HGT events. In this work, we evaluated the transfer of an ICE in physiological conditions mimicking those of the human GIT. The species S. thermophilus was used as a donor in the ICE transfer assays, as it is a food bacterium that can exchange genes with the resident microbiota during its transit in the GIT. As mentioned above, this species naturally harbors an ICE of the ICE*St3-*Tn*916* superfamily, whose transfer has been demonstrated previously *in vitro* (25). There is still debate about the ability of this species to survive in the GIT (29), so we tested two species as recipients, S. thermophilus and E. faecalis, an uncontested inhabitant of the GIT. Two strains of S. thermophilus, which differ in their resistance to stresses (acid pH, H_2_0_2_, bile salts) that prevail in the digestive tract (30), were used (either as donor or recipient). Experiments were first carried out in two of the most relevant dynamic *in vitro* systems, allowing mirroring of the human upper GIT for the first model (TNO gastro-Intestinal tract Model 1 [TIM-1]) and the large intestine for the second model (ARtificial COLon model [ARCOL]) (31). Studying gene transfer occurring in the small intestine is interesting since it is the first region where food bacteria come into contact with intestinal bacteria, and this GIT compartment is particularly rich in streptococci due to its communication with the oral cavity (32). The TIM-1 system has been developed and used for many years in various applications, in particular, to study survival and behavior of microorganisms such as probiotics and pathogens in the upper GIT (33–35). The ARCOL system enables the maintenance of the human gut ecosystem and metabolically active microbiota while reproducing the main physicochemical parameters of the human gut, i.e., temperature, pH, anaerobiosis, nutrient availability, and retention time (36, 37). Dynamic GIT models such as the TIM-1 and the ARCOL are increasingly used as an alternative to *in vivo* assays. They display advantages, among which accuracy, reproducibility, and cost-efficiency are often cited. In our case, they also gave us the opportunity to easily study the impact of different digestive environments along the human GIT on gene transfer between bacteria. Experiments were also carried out *in vivo* using a germfree mouse model. The mouse model has already been frequently used for *in vivo* gene transfer experiments since the histological structure of the GIT of rodents has similarities to the human gut, including epithelial layers and mucous secretion. Since the gut has natural resistance to colonization by foreign bacterial strains, the experiments were performed using germfree mouse. This model allows high colonization of inoculated bacteria. Thus, bacteria that are normally only transient (which is likely the case for S. thermophilus) will be able to colonize the GIT in high numbers due to the lack of competition with an indigenous microbiota. High numbers of bacteria will increase the chance of cell-cell contacts required for conjugation and thus increase the opportunity for gene transfer events.

## RESULTS

### ICE transfer in a simulated human upper gastrointestinal environment.

**FIG 1:**
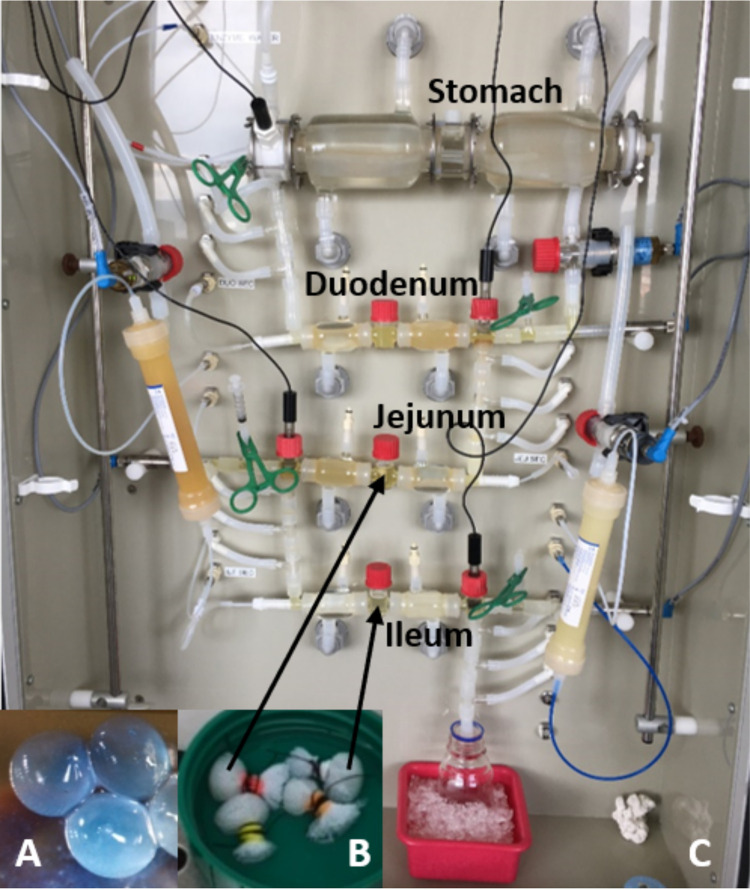
Pictures of bacterial cells entrapped in alginate, agar, and chitosan beads (A), nylon bags containing 50 beads (B), and the whole TIM-1 system with its different compartments (C).

To ensure the physical contact between donor and recipient strains that is required for the conjugation process, cells were entrapped in alginate, agar, and chitosan beads coated with chitosan (Fig. 1). Coating was necessary to prevent erosion during dynamic *in vitro* digestion. Nylon bags containing 50 beads were then easily placed in two different digestive compartments (jejunal and ileal) of the TIM-1 system (Fig. 1). Preliminary studies indicated that the survival of the cells was not impacted by this entrapment and that a high number of transconjugants were obtained when the beads were incubated in LM17 growth medium (data not shown).

**FIG 2:**
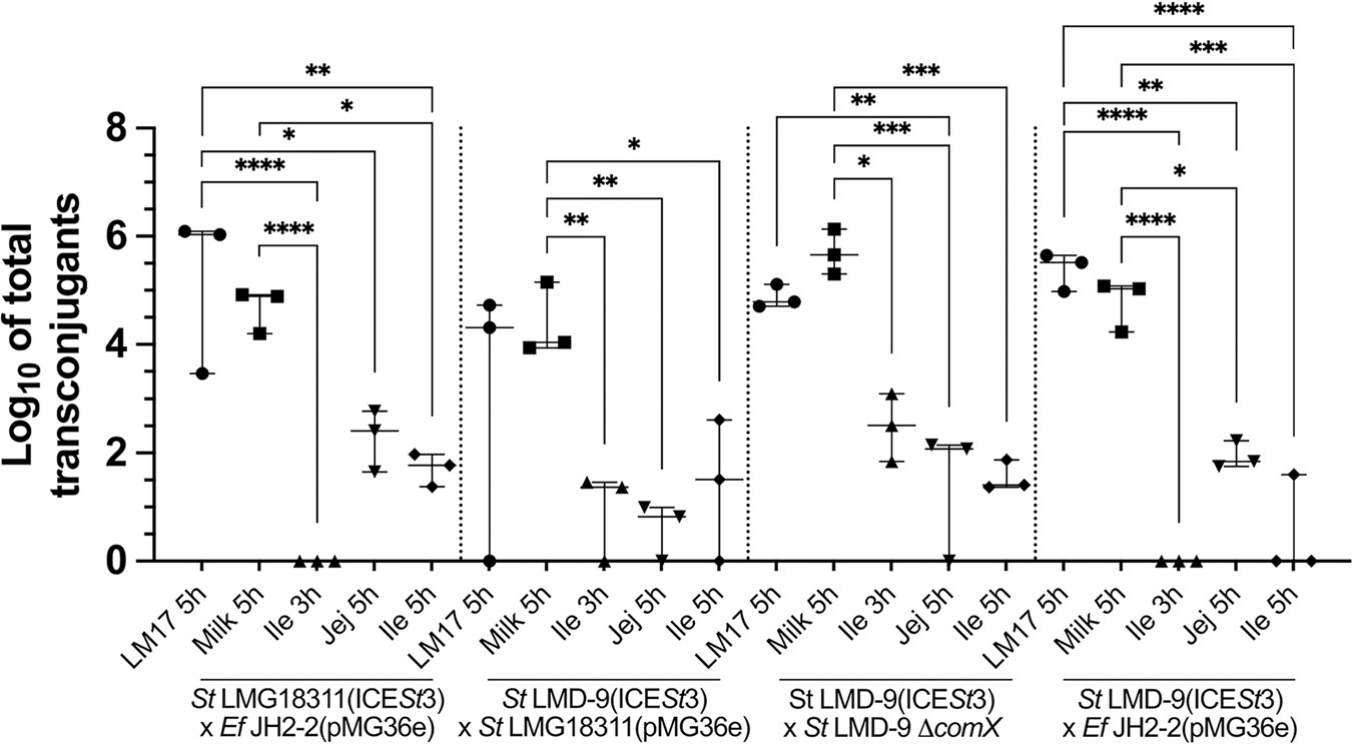
Total transconjugants numerated in 50 beads after a 5-h incubation in LM17 and milk (controls) and after 3- and 5-h incubations into the jejunum and the ileum compartments of the TIM-1 system during *in vitro* milk digestion. Bacteria entrapped in the beads were in the range of 2 × 10^6^ to 15 × 10^6^ and of 0.7 to 11 × 10^6^ CFU per bead for donors and recipients, respectively. The data obtained after a 3-h incubation in jejunum are not shown in the figure since no transconjugant was detected, whatever the mating pair tested. Median, minimum, and maximum values of three independent experiments are given. When the differences between two conditions are statistically significant, they are indicated by one to four stars (*, *P* < 0.05; **, *P* < 0.01; ***, *P* < 0.001; and ****, *P* < 0.0001), as determined by one-way ANOVA. Abbreviations: Ile, ileum; Jej, jejunum.

A first experiment using the TIM-1 was carried out using S. thermophilus LMG18311(ICE*St3*) as donor strain (as this strain was previously successfully used in *in vitro* conjugation assays [25, 38]) and E. faecalis JH2-2(pMG36e) as recipient (Table 1). Control experiments were made in parallel by incubating the beads in LM17 broth and milk. Both donor and recipient strains grew well in these control conditions, but their survival was reduced when placed in the ileal and jejunal compartments of the TIM-1 (Table 2). After 5 h of incubation in the jejunal compartment, less than 5% of S. thermophilus LMG18311(ICE*St3*) cells and 50% of E. faecalis JH2-2(pMG36e) cells had survived (Table 2). Despite the low survival of the strains, transconjugants were obtained in the jejunum and ileum but only after 5 h of incubation (Fig. 2; Table 3). In a second series of experiments, considering the low survival observed for S. thermophilus LMG18311 in the first experiment, we used S. thermophilus LMD-9(ICE*St3*) as donor strain because this strain was shown to better resist the physicochemical conditions encountered in the human digestive ecosystem (30, 35). Three strains were tested as recipients, (i) S. thermophilus LMG18311(pMG36e), (ii) S. thermophilus LMD-9 Δ*comX*, and (iii) E. faecalis JH2-2(pMG36e). In controls (incubation in LM17 broth and milk), all strains grew well, but, as in the first experiment, survival was highly impacted when beads were placed in the jejunal or ileal compartment of the TIM-1 (Table 2). In the jejunum, as in the first experiment, no transconjugant was recovered after 3 h of incubation, whatever the mating pair, but was obtained after 5 h (Fig. 2; Table 3) despite a sharp decrease in survival rates of the donor and recipients (less than 3% for all S. thermophilus strains and 30% for E. faecalis JH2-2 at 5 h) (Table 2). For all mating pairs, the number of transconjugants obtained in this compartment is not significantly different from the one obtained after 5 h of incubation in the ileum (even though the bacterial survival was better in this compartment) (Fig. 2; Tables 2 and 3). Moreover, S. thermophilus LMD-9(ICE*St3*), combined with either S. thermophilus LMG18311(pMG36e) or S. thermophilus LMD-9 Δ*comX*, was able to give transconjugants in the ileum after 3 h of incubation at similar levels to the ileum and jejunum at 5 h (Fig. 2; Table 3).

**TABLE 1:** Bacterial strains and plasmid used in this study

Strain or plasmid	Relevant phenotype(s)^*a*^	Source(s) or reference
Strains		
Streptococcus thermophilus		
LMG18311	Wild-type strain	BCCM, LMG
LMD-9	Wild-type strain	ATCC BAA-491
LMG18311(ICE*St3*)	LMG18311 carrying ICE*St3* tagged with a chloramphenicol resistance cassette (Cm^r^)	25
LMG18311(pMG36e)	LMG18311 carrying the pMG36e plasmid conferring Ery^r^	25
LMD-9(pMG36e)	LMD-9 carrying the pMG36e plasmid conferring Ery^r^	38
LMD-9(ICE*St3*)	LMD-9 carrying ICE*St3* tagged with a chloramphenicol resistance cassette (Cm^r^; derived from LMD-9(ICE*St3*, pMG36e) after curation of the pMG36e plasmid)	38, this work
LMD-9 Δ*comX*	LMD9 strain deleted of the *comX* gene (encoding the sigma X factor involved in competence development) by insertion of an erythromycin resistance cassette (Ery^r^)	This work
Enterococcus faecalis		
JH2-2(pMG36e)	JH2-2 strains carrying the pMG36e plasmid conferring erythromycin resistance, Rif^r^, Fus^r^, Ery^r^	50
Plasmids		
pMG36e	3.6 kb, replication origin from pWV01; Ery^r^	53
pGhost9-Ery	3.7 kb, replication origin from pWV01; Ery^r^	54

a Cm^r^, chloramphenicol resistance; Ery^r^, erythromycin resistance; Rif^r^, rifampin resistance; Fus^r^, fusidic acid resistance.

**TABLE 2:** Survival rates (no. [%]) of donor and recipient strains in beads after 3- and 5-h incubations into the jejunum and the ileum compartments of the TIM-1 system during *in vitro* milk digestion and after a 5 h-incubation in LM17 and milk (controls run in parallel of the *in vitro* digestion)^*a*^

Donor × recipient	Survival rate (no. [%] of survivors) after incubation in:											
	LM17 (5 h)		Milk (5 h)		TIM jejunum (3 h)		TIM ileum (3 h)		TIM jejunum (5 h)		TIM ileum (5 h)	
	Donor	Recipient	Donor	Recipient	Donor	Recipient	Donor	Recipient	Donor	Recipient	Donor	Recipient
S. thermophilus LMG18311(ICE*St3*) × E. faecalis JH2-2(pMG36e)	399 (347)	1,681 (8,921)	617 (869)	1,186 (604)	2 (9)	49 (358)	23 (6)	80 (354)	3 (10)	18 (129)	12 (12)	44 (944)
S. thermophilus LMD-9(ICE*St3*) × S. thermophilus LMG18311(pMG36e)	675 (2,071)	983 (943)	2,518 (3,481)	530 (493)	12 (9)	1.5 (7)	59 (45)	37 (28)	2 (2)	0.03 (1)	24 (32)	5 (18)
S. thermophilus LMD-9(ICE*St3*) × S. thermophilus LMD-9 Δ*comX*	2,148 (1,997)	967 (2,020)	362 (3,354)	2,733 (8,695)	4 (4)	7 (9)	47 (29)	74 (44)	3 (9)	1 (8)	12 (11)	20 (10)
S. thermophilus LMD-9(ICE*St3*) × E. faecalis JH2-2(pMG36e)	1,161 (654)	830 (670)	910 (1,138)	125 (151)	3 (21)	104 (29)	32 (14)	120 (41)	1 (2)	30 (12)	8 (14)	86 (40)

a Beads were rinsed and mechanically disrupted in a saline solution to liberate bacteria and to allow their enumeration by plating on selective medium (either chloramphenicol 8 μg mL^−1^ for donor or erythromycin 10 μg mL^−1^ for recipient). Entrapped bacteria were in the range of 2 × 10^6^ to 15 × 10^6^ and of 0.7 × 10^6^ to 11 × 10^6^ CFU per bead for donors and recipients, respectively. Percentages were obtained by comparing the number of CFU per bead at each time of the experiment with the number of CFU of bacteria per bead at the initial time. A percentage higher than 100% indicates a multiplication of the bacteria, whereas a percentage lower than 100% indicates death of the bacteria. Results obtained for three independent experiments are indicated as medians with interquartile ranges indicated in brackets.

**TABLE 3:** Total transconjugants (Tc) enumerated in 50 beads after 3 and 5-h incubations into the jejunum and the ileum compartments of the TIM-1 system during *in vitro* milk digestion or after 5-h incubation in LM17 and milk (controls run in parallel of the *in vitro* digestions)^*a*^

Donor × recipient	Total Tc (CFU) in:					
	LM17 (5 h)	Milk (5 h)	TIM-1 jejunum (3 h)	TIM-1 ileum (3 h)	TIM-1 jejunum (5 h)	TIM-1 ileum (5 h)
S. thermophilus LMG18311(ICE*St3*) × E. faecalis JH2-2(pMG36e)	1.1 × 10^6^ (6.2 × 10^5^)	7.8 × 10^4^ (3.4 × 10^4^)	0 (0)	0 (0)	2.6 × 10^2^ (2.8 × 10^2^)	6.0 × 10^1^ (3.6 × 10^1^)
S. thermophilus LMD-9(ICE*St3*) × S. thermophilus LMG18311(pMG36e)	2.1 × 10^4^ (2.7 × 10^4^)	1.1 × 10^4^ (6.6 × 10^4^)	0 (0)	2.3 × 10^1^ (1.4 × 10^1^)	6.7 × 10^0^ (5.0 × 10^0^)	3.3 × 10^1^ (2.1 × 10^2^)
S. thermophilus LMD-9(ICE*St3*) × S. thermophilus LMD-9 Δ*comX*	6.2 × 10^4^ (3.9 × 10^4^)	4.5 × 10^5^ (5.8 × 10^5^)	0 (0)	3.2 × 10^2^ (5.9 × 10^2^)	1.2 × 10^2^ (7.0 × 10^1^)	2.6 × 10^1^ (2.6 × 10^1^)
S. thermophilus LMD-9(ICE*St3*) × E. faecalis JH2-2(pMG36e)	3.3 × 10^5^ (1.8 × 10^5^)	1.1 × 10^5^ (5.2 × 10^4^)	0 (0)	0 (0)	7.1 × 10^1^ (5.7 × 10^1^)	0 (2.0 × 10^1^)

a Beads were rinsed and mechanically disrupted in a saline solution to liberate bacteria and to allow their enumeration by plating on selective medium (5 μg mL^−1^ chloramphenicol and 8 μg mL^−1^ erythromycin). Entrapped bacteria were in the range of 2 × 10^6^ to 15 × 10^6^ and of 0.7 × 10^6^ to 11 × 10^6^ CFU per bead for donors and recipients, respectively. Results obtained for three independent experiments are indicated as medians with interquartile ranges indicated in brackets.

In addition, we investigated the presence of viable bacteria outside the beads during TIM-1 experiments (Table S1 in the supplemental material). Transconjugants were not detected in ileal effluents or in the final jejuno-ileal content during all TIM-1 experiments. Among recipient strains, only E. faecalis JH2-2(pMG36e) was recovered from ileal effluents and final jejuno-ileal content, whereas very few cells of donors were enumerated during the 5 h of *in vitro* digestion (Table S1).

### ICE transfer in the simulated human colonic environment.

**FIG 3:**
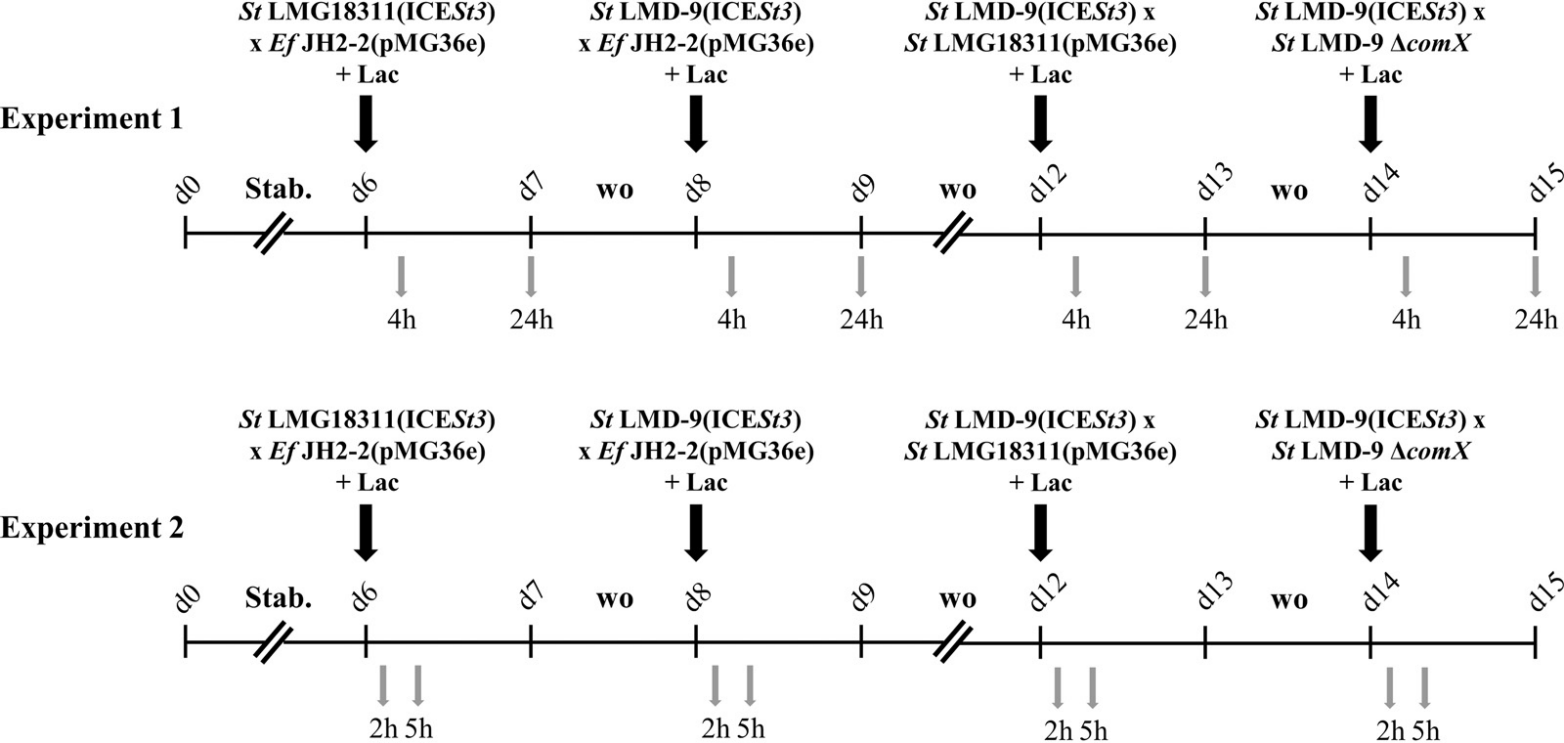
Timeline of the *in vitro* experiments in ARCOL investigating ICE*St3* transfer with four donor-recipient mating pairs. ARCOL was inoculated with fresh stools from healthy volunteers at day 0 (a 50-year-old man for experiment 1 and a 27-year-old woman for experiment 2). After a stabilization period (Stab.) of 6 days, two bags containing 50 beads of entrapped donor-recipient pair were introduced in the system. Lactose (Lac) was added at 2 g/L at the same time. Bags were maintained in the colonic environment for 4 and 24 h in the first experiment and for 2 and 5 h in the second one. The four mating pairs were tested successively after at least a 1-day washout period (wo). Abbreviations: *St*, Streptococcus thermophilus; *Ef*, Enterococcus faecalis.

The four donor-recipient mating pairs that gave transconjugants during *in vitro* digestion of milk in the TIM-1 system were tested in the ARCOL model simulating the luminal environment of healthy adult colon in a bioreactor. Two independent experiments with different microbial gut communities coming from the feces of two healthy donors were run. Bioreactors successively housed the four selected mating pairs (2 bags of 50 beads) for a maximum of 24 h (Fig. 3). Survival rates of all bacteria in the beads were evaluated (Table S2). E. faecalis JH2-2 and S. thermophilus LMD-9 Δ*comX* used as recipients were not impacted after a 4- to 5-h incubation in the colonic environment. S. thermophilus LMD-9(ICE*St3*) also resisted (up to 43% of survival after 4 to 5 h), whereas S. thermophilus LMG18311 used as recipient or as donor poorly survived (1 to 8% of survival after 4 to 5 h). In this context, only one clone displaying the expected resistance phenotype was obtained in beads after 5 h of coincubation of S. thermophilus LMG18311(ICE*St3*) and E. faecalis JH2-2(pMG36e) during the second experiment. This clone was confirmed to be a transconjugant by PCR.

### *In vivo* animal studies.

Two donor-recipient pairs [S. thermophilus LMD-9(ICE*St3*)-S. thermophilus LMD-9 Δ*comX* and S. thermophilus LMD-9(ICE*St3*)-E. faecalis JH2-2] that gave transconjugants in the TIM-1 experiment were selected for *in vivo* conjugation experiments using a mouse animal model. Preliminary experiments indicated that S. thermophilus LMD-9 colonizes the mice at a higher level than S. thermophilus LMG18311 (data not shown), so this strain was chosen for the *in vivo* assays. In addition, in preliminary *in vivo* experiments, the pMG36e plasmid hosted by the recipient S. thermophilus strains was lost after a few days, so the strain with a chromosomal resistance marker was chosen as recipient (S. thermophilus LMD-9 Δ*comX*). This strain is deficient in competence, so only the acquisition of genes by conjugation will be measured.

**FIG 4:**
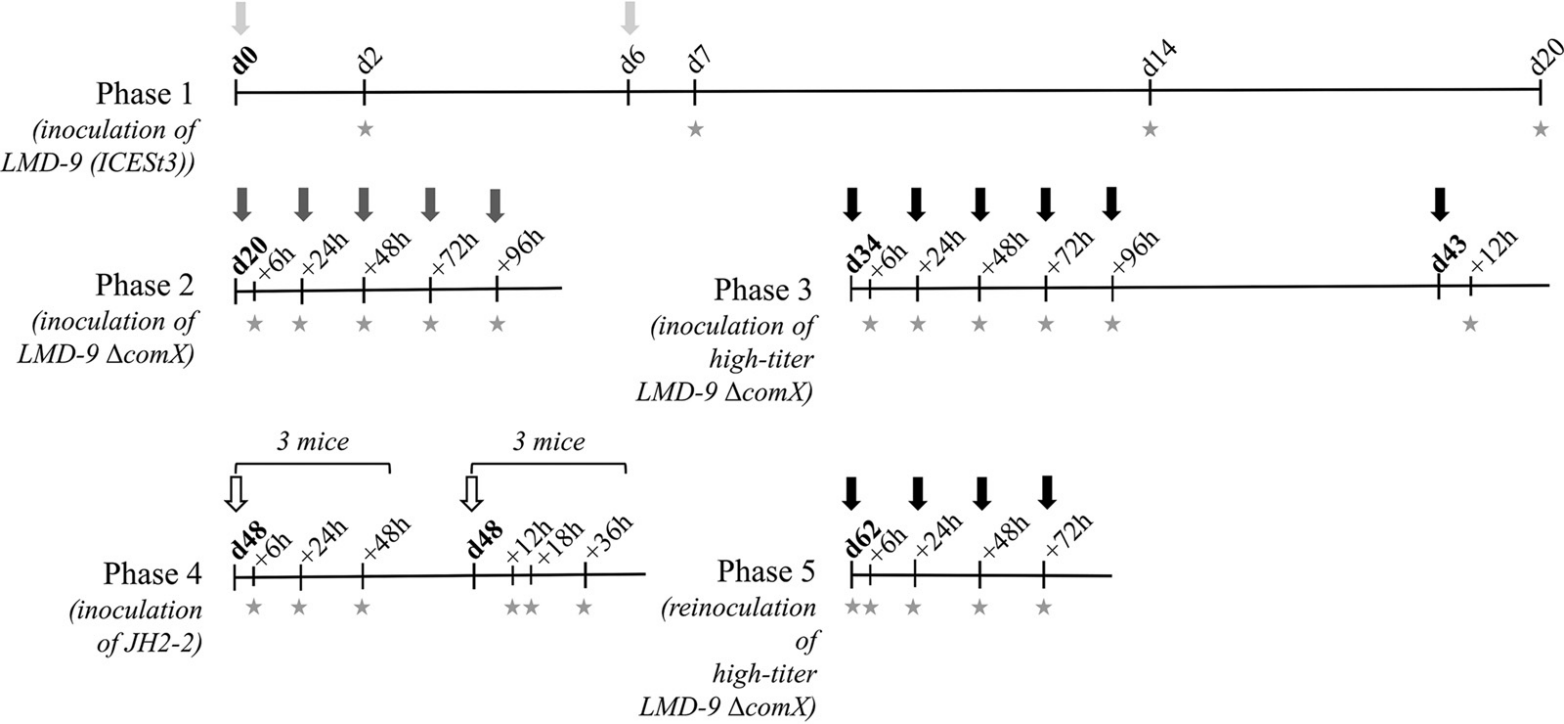
Timeline of the *in vivo* experiments investigating ICE transfer from the donor strain S. thermophilus LMD-9(ICE*St3*) to the recipient strains S. thermophilus LMD-9 Δ*comX* and E. faecalis JH2-2(pMG36e). Phase 1, monoassociation of the mice (*n *= 6) by inoculation of the S. thermophilus donor strain (light-gray arrow); phase 2, inoculation of the S. thermophilus recipient strain (dark-gray arrow); phase 3, inoculation of the S. thermophilus recipient strain, using a 20 times more concentrated inoculum (black arrow); phase 4, inoculation of the E. faecalis recipient strain (white arrow); phase 5, inoculation of the S. thermophilus recipient strain (black arrow) in the presence of the E. faecalis strain. Stars indicate times of feces collection for bacterial cell enumeration.

**FIG 5:**
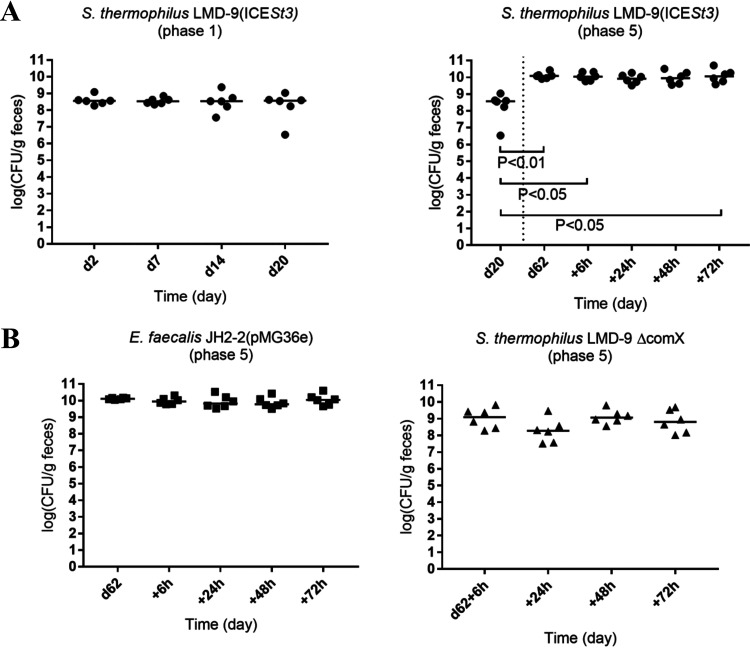
Fecal concentrations of the strains throughout the *in vivo* experiment. (A) Fecal concentrations of the donor strain S. thermophilus LMD-9(ICESt3) in phases 1 and 5. Within phase 1, concentrations at day 7, day 14, and day 20 were compared to the concentration achieved at day 2, i.e., 2 days after inoculation. Within phase 5, concentrations were compared to day 20, taken as the reference point of S. thermophilus LMD-9(ICESt3) establishment in the gut. The nonparametric Friedman test for repeated measures, followed by Dunn’s multiple-comparison test, was used. Only significant differences (*P *< 0.05) are indicated. (B) Fecal concentrations of the recipient strains E. faecalis JH2-2(pMG36e) and S. thermophilus LMD-9 Δ*comX* in phase 5. For each strain, concentrations at the different time points were not significantly different. The nonparametric Friedman test for repeated measures, followed by Dunn’s multiple-comparison test, was used. Only significant differences (*P *< 0.05) are indicated.

After optimization of the protocol of inoculation of the strains to the mouse (successive and repetitive strain inoculation if necessary; see Fig. 4 for more details), all strains (donor and recipient) successfully colonized the mouse at a high level [interquartile range (IQR), 9.9 to 10.2 log(CFU/g) for S. thermophilus LMD-9(ICE*St3*); interquartile range, 7.6 to 8.8 log(CFU/g) for S. thermophilus LMD-9 Δ*comX*; and interquartile range, 10.1 to 10.2 log(CFU/g) for E. faecalis JH2-2 at the beginning of phase 5] (Fig. 5).

However, despite these high levels of mouse colonization by the donor and recipient strains, no transconjugant (either with the recipient strain S. thermophilus LMD-9 Δ*comX* or with the recipient strain E. faecalis JH2-2) was isolated at any phase of the protocol.

## DISCUSSION

Genome sequence analysis supports the hypothesis that the GIT is conducive to gene transfers between various bacterial species. However, only a few studies examined the conjugative transfer of mobile genetic elements, in particular, ICEs, in physiological conditions close to those encountered in the human GIT (39–42).

In this work, we used three different systems mimicking these physiological conditions to detect *in situ* HGT events occurring by conjugation, including (i) an artificial digester that mimics the upper part of the digestive tract (TIM-1 system), (ii) an artificial colon (ARCOL system), and (iii) a mouse model.

The conjugative element studied in this work, ICE*St3*, initially described in S. thermophilus, does not encode any adhesion protein or pili that could help ensure the physical contact between donor and recipient cell that is required for DNA conjugative transfer. As a consequence, no conjugative transfer of this ICE is detectable in liquid medium (25). To increase the likelihood of conjugative transfer, we thus entrapped the mating pairs in alginate, agar, and chitosan beads and placed them in different intestinal compartments in the TIM-1 systems. The use of chitosan both facilitates cell-cell contacts required for conjugation and offers the bacteria protection against adverse physiological conditions found in the upper GIT. In natural settings, such protection can also be provided by the food matrix. Despite the protection offered by entrapment in beads, the survival of the strains was highly impacted by the adverse conditions encountered in the jejunal and ileal compartments of the TIM-1 (Table 2). This sharp decrease in survival of the strains and the differential impact on the growth rate of the incubation conditions preclude any calculation and comparison of ICE transfer frequencies based on population densities. Thus, only the absolute number of transconjugants per 50 beads determined in the various incubation conditions was considered a valuable indicator.

Transconjugants were obtained with all the mating pairs tested during milk digestion in the TIM-1 system. For these reasons, the TIM-1 system appears interesting to evaluate the acquisition of foreign genes through food consumption. The number of transconjugants was systematically lower in the TIM-1 (whatever the compartment) than in the control conditions (LM17 or milk). This likely correlates with the poor survival of the strains in the TIM-1 compartments that directly impacts the donor and recipient initial population density. A 5-h incubation of beads in the jejunum was the sole condition that enabled the obtention of transconjugants for all the mating pairs tested. Thus, this condition appears as the most propitious for transconjugant observation. In the jejunum, bile salt concentration is higher than in the ileum. This high-stress exposure could have an impact on ICE activation and transfer. This also indicates that the obtention of transconjugants does not correlate with the strains’ survival since the 5-h incubation in jejunum is responsible for the highest decrease in strains’ survival.

In contrast to the multiple clones of transconjugants obtained in the TIM-1 system, only one clone was obtained in the artificial colon. As in the TIM-1 system, donor and recipient cells were protected and in close contact with each other by entrapment in alginate, agar, and chitosan beads. However, the physiological conditions encountered in the colon largely differ from those encountered in the upper GIT. The most plausible explanation for the difference in gene transfer observed between the two GIT compartments could be a higher induction of conjugative transfer in the upper GIT due to the stresses encountered (bile salts in particular).

No transfer was detectable in the mouse model. As mentioned previously, the protocol used was optimized in order to reach high colonization levels of both the donor and recipient strains. However, compared to the artificial systems, bacterial cells were not entrapped in alginate, agar, and chitosan beads. In the artificial systems, entrapment of bacteria in beads ensured close cell-cell contacts. Conjugation in the gut microbiota was proposed to occur in the mucus layer of the intestinal lumen after biofilm formation, thus providing a relatively stable matrix akin to an agar plate or gel bead used for the *in vitro* conjugation experiments. However, almost all conjugative plasmids tested until now displayed a lower transfer *in vivo* than if tested *in vitro* (43). For plasmids that lack mating pair stabilization, the frequency was very low or even undetectable (44). In our assays, donor and recipient strains were inoculated separately in order to obtain good colonization of the mice by all the strains. Thus, it is possible that the mixing was not sufficient to ensure the physical contact required for conjugation between the donor and recipient cells. The search for transconjugants was done using feces during the experiment and in cecal content at the end of the experiment, not in the mucus layer. Although we cannot completely exclude that transconjugants were missed, this seems unlikely since there has been no demonstration of S. thermophilus adhesion to mucus until now (43).

The conditions used in this work were restricting since we used only a limited number of donor and recipient strains. In real physiological conditions, the richness and diversity of the bacterial gut community would offer more opportunities for HGT events to occur. In addition, several factors potentially increasing *in vivo* HGT efficiency in the intestine were identified, including SOS-inducing agents, stress hormones, microbiota, and microbiota-derived factors (14). Furthermore, we used a simplified model involving a single conjugative element present in the donor strain whose transfer is not optimal even *in vitro* (maximal frequency of transfer observed of 10^−4^ transconjugant per donor cell) (38). This is far from the natural conditions where MGEs display complex relationships (45).

Even if HGT events are very rare, expansion of transconjugants can happen if ecological success is fostered by selecting conditions or by events that destabilize the microbial community and facilitate the establishment of exogenic bacteria. In addition, MGEs have their own distinct parameters (including ecological ones) of persistence and multiplication after transfer (46–48).

In conclusion, an intraspecies and an interspecies (toward E. faecalis) transfer of ICE*St3*, an ICE of the Tn*916* superfamily, was obtained in conditions mimicking the human upper digestive tract. E. faecalis appeared to be a good candidate for foreign gene acquisition. The enumeration of bacteria in the ileal and jejunal effluents indicated that it was able to survive outside the beads. This is not surprising since this species is known to resist high titers of bile salts (exploited for its specific isolation on bile esculin selective medium). Due to its high ability to colonize the gut and acquire MGEs, this commensal bacterium could serve as an intermediate in gene transfers and as a reservoir of genes for other species in the gut. The TIM-1 system appears as an interesting tool to evaluate HGT events in conditions closer to the digestive tract than on agar plates. However, most of the gene transfers likely occur in the colon where the microbial community is very rich and diverse. The complexity of this ecosystem makes it difficult to detect rare HGT events. The culture-based approach is probably not the most appropriate method to detect such events, and molecular methods (in particular, single-cell time-lapse fluorescence methods) would likely be more sensitive.

## MATERIALS AND METHODS

### Bacterial strains and culture conditions.

The strains used in this study are listed in Table 1. Due to frequent plasmid loss during *in vivo* experiments, we constructed a recipient strain labeled with a chromosomal antibiotic resistance cassette. In order to avoid DNA acquisition by natural transformation that would interfere with the measurement of ICE transfer by conjugation, the *comX* locus was chosen as the target for labeling the recipient strain. This locus encodes a specific sigma factor required for the transcription of late competence genes in S. thermophilus (49). The LMD9 Δ*comX* mutant was constructed by natural transformation of the LMD9 strain using an overlap PCR product. This amplicon was obtained by joining three PCR products (including an erythromycin resistance cassette) as described previously (50). Two PCR fragments were obtained by PCR amplification of genomic DNA of strain S. thermophilus LMD-9 using primers comX-1 LMD9 (GACGTAGTAGAGTTGGCGTTCC), comX-2-ery LMD9 (GCACTATCAACACACTCTTAAGTTTGCTTCTTGTTCCATTGAACCTCC), comX-3-ery LMD9 (CCAAGGAGCTAAAGAGGTCCCCATGTAATGAAGAAGACTGAG), and comX-4 LMD9 (GGTTTGTGGCTGTGTTTTCAAATG). The third fragment corresponding to the erythromycin resistance cassette was obtained by PCR amplification of pGhost9-ery using primers Empg 9F (CAAACTTAAGAGTGTGTTGATAGTGC) and Empg 9R (GGACCTCTTTAGCTCCTTGG). Plasmid curing of the LMD9(ICE*St3*, pMG36e) was done as described previously (50).

For the mating experiments, several strains were used as donor strains [S. thermophilus LMG18311(ICE*St3*) and S. thermophilus LMD-9(ICE*St3*)] and as recipient strains [S. thermophilus LMG18311(pMG36e), S. thermophilus LMD-9 Δ*comX*, and E. faecalis JH2-2(pMG36e)].

S. thermophilus (strains LMG 18311 and LMD-9 and their derivatives) and E. faecalis JH2-2 were routinely grown in M17 broth supplemented with 0.5% lactose (LM17) and adequate antibiotic (5 μg mL^−1^ chloramphenicol for donor strains or 5 μg mL^−1^ erythromycin for recipient strains) at 42°C for S. thermophilus strains or 37°C for the E. faecalis strain without shaking. For strain enumeration during TIM-1 experiments, adequate dilutions in saline solution (9 g L^−1^ NaCl) were plated (or included) on LM17 agar supplemented with antibiotics at the following concentrations: 8 μg mL^−1^ chloramphenicol for donor strains, 10 μg mL^−1^ erythromycin for recipient strains, and 5 μg mL^−1^ chloramphenicol and 8 μg mL^−1^ erythromycin for transconjugant selection. Plates were incubated aerobically for 24 h. In ARCOL experiments, the same conditions were used to enumerate donors and recipients, except for E. faecalis JH2-2(pMG36e), for which 50 μg mL^−1^ rifampin was combined with 10 μg mL^−1^ erythromycin. Transconjugants were selected on LM17 agar containing 8 μg mL^−1^ chloramphenicol and 10 μg mL^−1^ erythromycin, supplemented with 50 μg mL^−1^ rifampin when E. faecalis JH2-2 was used as recipient. No bacteria from artificial colonic contents were able to grow on LM17 agar supplemented with chloramphenicol and erythromycin and, eventually, rifampin.

The inoculum used for animal studies was prepared by growing cells overnight at 37°C in M17 broth supplemented with glucose (1%) for S. thermophilus strains and in bile esculin sodium azide broth (BEA) for E. faecalis. The cultures were pelleted by centrifugation and washed twice in phosphate-buffered saline (PBS), and dry pellets were stored at −80°C until use. The bacterial cell pellets were extemporaneously thawed and resuspended in PBS or skimmed milk on the day of inoculation.

Enumeration of bacterial cells in feces of mice was done by plating serial dilutions of fresh feces on M17 agar supplemented with either chloramphenicol (8 μg mL^−1^) for S. thermophilus LMD9(ICE*St3*), erythromycin (10 μg mL^−1^) for S. thermophilus LMD9 Δ*comX*, or both antibiotics, chloramphenicol (4 μg mL^−1^) and erythromycin (5 μg mL^−1^), for S. thermophilus transconjugants (i.e., clones obtained after transfer of the ICE to recipient cells). Similarly, E. faecalis cells were enumerated by plating serial dilutions on BEA agar with rifampin (25 μg mL^−1^) for E. faecalis JH2-2(pMG36e) or with chloramphenicol (4 μg mL^−1^) for E. faecalis transconjugants. Agar plates were incubated at 37°C, and colonies were counted after 24 h of incubation.

### *In vitro* conjugation experiments.

**(i) Encapsulation of mating pairs in beads.** As conjugation does not occur if strains are not in direct contact, donor and recipient strains were encapsulated in alginate, agar, and chitosan beads as follows. The following solutions for encapsulation were prepared in demineralized water and sterilized at 121°C for 15 min: 3% agar (Bacto Agar; BD-Difco, Sparks, MD, USA), 4% sodium alginate (catalog no. W201502; Sigma-Aldrich, St-Louis, MO, USA), 0.4 M calcium chloride dihydrate, and 0.8% chitosan (catalog no. 428851000; Acros Organics, NJ, USA) dissolved in 0.5% acetic acid solution.

Donor and recipient strains were grown overnight in 90 mL of LM17 broth at 37°C supplemented with appropriate antibiotic for each one. Cultures were centrifuged at 3,200 × *g* for 5 min at room temperature. Pellets were resuspended with 1.5 mL of sterile saline solution, and the two suspensions were mixed. Agar and alginate solutions were maintained at 50°C in a water bath before use. The bacterial suspension (3 mL) was mixed with 8.5 mL of alginate and then with 8.5 mL of agar. The mixture was quickly poured through a drop-by-drop system with a constant flow (2 mL/min) in 75 mL of calcium chloride-chitosan solution (50:50, vol/vol) at room temperature under soft magnetic stirring. Beads were maintained under stirring for 30 min and then transferred in 40 mL of 0.8% chitosan for coating (15 min under soft stirring). Beads were rinsed with saline solution before use. Enumeration of entrapped bacteria (donors and recipients) was realized before each *in vitro* experiment: 50 beads were mechanically disrupted (13,500 rpm; Ultra-Turrax; IKA) in 10 mL of saline solution for 45 s to liberate bacteria, and then, the bacterial suspension was processed for serial dilution before plating. Entrapped bacteria were in the range of 2 × 10^6^ to 15 × 10^6^ and of 0.7 × 10^6^ to 11 × 10^6^ CFU per bead for donors and recipients, respectively.

For *in vitro* gastrointestinal digestions and colonic fermentations, nylon bags entrapping 50 beads per bag were prepared to allow maintenance of beads into the desired compartment and facilitate their sampling.

**(ii) Conjugation experiments in the TIM-1 dynamic system.** Donor-recipient pairs were tested in triplicate in three conditions, in the dynamic *in vitro* system TIM-1 and in control conditions where 50 beads were incubated in 50 mL of LM17 broth or 50 mL of commercial semiskimmed ultrahigh temperature (UHT) milk at 37°C and 100 rpm. The LM17 broth was the growth medium that was previously used to demonstrate that ICE*St3* is an active ICE (25). The second growth medium used, milk, was tested since it is closer to the “real-life conditions” of S. thermophilus. The TIM-1 system was set to simulate the digestion of 300 mL milk in a healthy human adult as described previously but with a slight modification (2-fold pepsin concentration) (35). Two nylon bags entrapping 50 beads each were maintained, respectively, in the jejunal and ileal compartments of the TIM-1. Digestion and controls were run for 5 h. One bag per digestive compartment was withdrawn for bacteria enumeration into beads after 3 h and the second one at the end of the digestion. Bacteria were also enumerated in ileal effluents collected from hour to hour and the final jejuno-ileal content of the TIM-1 to see if they can be released from the beads and survive freely in the intestinal environment. Enumeration in controls was done on beads after 5 h of incubation. Collected beads were rinsed with saline solution, and enumeration of donors, recipients, and transconjugants proceeded as described above. Enumeration of free bacteria was realized on appropriate dilutions of ileal effluents and final jejuno-ileal content.

At least 10 clones were confirmed to be transconjugants by PCR targeting the integrase gene of ICE*St3* and the pMG36e plasmid as described previously (50) or the erythromycin resistance cassette for the LMD-9 Δ*comX* recipient strain.

**(iii) Conjugation experiments in ARCOL.** The behavior of encapsulated bacteria and transconjugant formation in the colonic environment was evaluated *in vitro* using the ARCOL model described by Deschamps and colleagues (37) but without reproduction of the mucus-associated microbiota. Indeed, the module used to contain mucin beads was used in the present work to entrap our alginate, agar, and chitosan beads containing donor-recipient pairs. Briefly, ARCOL is a one-stage fermentation bioreactor (MiniBio; Applikon, Delft, The Netherlands) used under continuous conditions where pH (6.3), temperature (37°C), stirring (400 rpm), and volume (300 mL) were controlled. A nutritive medium containing sources of complex carbohydrates, proteins, lipids, minerals, and vitamins (36) is introduced continuously to simulate a mean retention time of 24 h. Lactose (2 g/L) was also added into the bioreactor at the time of introducing beads, as it was essential for conjugation. Two experiments were conducted independently, inoculating bioreactors with fresh fecal samples from healthy human volunteers (a 27-year-old woman and a 50-year-old man) with no history of antibiotic treatment 3 months prior to the beginning of the experiments. Following a 6-day stabilization period, four donor-recipient pairs were tested successively following experimental designs described in the supplemental material (Fig. 3). Two bags containing 50 beads of donor-recipient pair was maintained for 4 and 24 h (first experiment) or for 2 and 5 h (second experiment), respectively (Fig. 3) into the colonic environment and then withdrawn to enumerate donors, recipients, and transconjugants by plating as described above.

### *In vivo* conjugation experiments in gnotobiotic mice.

**(i) Animals.** Three male and 3 female C3H/HeN germfree mice, 10 weeks old, were obtained locally from the germfree rodent breeding unit of Anaxem, the germfree animal facility of the Micalis Institute (INRAE, France; license number: B78-322-6). They were transferred in a flexible-film isolator (Getinge, Les Ulis, France), which was ventilated with HEPA-filtered sterile air under positive pressure. The isolator was fitted with a DPTE aseptic transfer system (Getinge) allowing sterile connection of containers (Getinge) to import sterile consumables and germfree mice. Inside the isolator, male and female mice were housed separately in collective cages (3 mice/cage) containing sterile bedding made of wood shavings. The living environment was enriched with shredding paper and gnawing wood sticks. The mice had free access to autoclaved tap water and a γ-irradiated (45 kGy) standard diet (R03; Scientific Animal Food and Engineering, Augy, France). The animal room was maintained at 20 to 24°C and kept on a 12-h light/dark cycle (lights on at 7:30 a.m.). The animals were examined daily to ensure that they stay healthy and were weighed at the end of the experiment to confirm and compare their growth.

**(ii) Design of animal studies.** The mice were individually identified with electronic chips (IntelliBio, Seichamps, France) implanted under the skin in the interscapular region. As the adaptive response of S. thermophilus in the gut of mono-associated rodents may be improved by lactose supplementation (51), lactose (4.5%, wt/vol) was added to the drinking water throughout the experiment.

The experiment was organized in 5 phases (Fig. 4). First, the mice were mono-associated with the S. thermophilus LMD-9(ICE*St3*) donor strain. For this purpose, they were inoculated intragastrically, using flexible gavage tubes, with 0.2 mL of a cell suspension of the strain (0.3 × 10^8^ CFU), twice at 1-week intervals. Fresh feces were then collected from each mouse at intervals for 3 weeks to monitor the establishment of the strain in the gut. Indeed, previous work has shown that S. thermophilus colonizes progressively the gut of germfree rodents to reach a stable population in 3 to 4 weeks (52).

The second phase consisted of studying ICE transfer from the S. thermophilus LMD-9(ICE*St3*) donor strain to the S. thermophilus LMD-9 Δ*comX* recipient strain. The mice were inoculated intragastrically with 0.2 mL of a cell suspension of the recipient strain (0.5 × 10^8^ CFU) five times at 24-h intervals. Fresh feces were collected regularly during this administration period to look for transconjugants.

As no transconjugant was detected in the second phase of the experiment, the third phase consisted of renewing the administration of the S. thermophilus LMD-9 Δ*comX* recipient strain, using a 20 times more concentrated inoculum (1.70 × 10^9^ to 2.60 × 10^9^ CFU) in order to increase the number of bacterial cells likely to receive ICE from the donor strain in the mouse gut.

In the fourth phase, the transfer of ICE from the S. thermophilus LMD-9(ICE*St3*) donor strain to E. faecalis JH2-2 was investigated by inoculating the mice with orogastric gavage with 0.2 mL of a cell suspension of the E. faecalis strain (4.5 × 10^8^ CFU) once. Then, feces were collected at intervals for 2 days to monitor the establishment of E. faecalis in the gut and to look for E. faecalis transconjugants.

The fifth phase consisted of studying ICE transfer from the S. thermophilus LMD-9(ICE*St3*) donor strain to the S. thermophilus LMD-9 Δ*comX* recipient strain in the presence of E. faecalis JH2-2. Indeed, we wanted to test if the E. faecalis strain may serve as an intermediate recipient that can assist ICE transfer from the S. thermophilus donor strain to the S. thermophilus recipient strain. For this purpose, the mice were reinoculated with S. thermophilus LMD-9 Δ*comX*, and fresh feces were collected according to the same protocol as in the third phase.

One day after the end of the fifth phase, the mice were killed by cervical dislocation, and cecal contents were collected to enumerate the three strains and the transconjugants.

All procedures were carried out in accordance with the European guidelines for the care and use of laboratory animals and approved by the ethics committee of the INRAE Research Centre at Jouy-en-Josas (approval reference, APAFIS no. 1234-2015101315238694 v1).

### Calculations and statistical analyses.

For *in vitro* studies, the survival rate of donor and recipient strains in beads was calculated as the percentage of viable bacteria enumerated after 3 or 5 h of incubation in TIM-1 compartments or in controls compared to initial amounts. Transconjugants obtained in controls and TIM-1 were expressed as the median of total CFU/50 beads or log(CFU/50 beads) from three independent experiments. The interquartile range (IQR) was calculated as the difference between quartile 3 and quartile 1. Comparisons were performed using the one-way analysis of variance (ANOVA) test. The level of significance was set at a *P* value of <0.05. For animal studies, fecal concentrations of the bacterial strains were expressed as log(CFU/g feces). For S. thermophilus LMD-9(ICE*St3*), concentrations were compared within phase 1. As no difference occurred, we took the concentration at the end of this phase as the reference point and compared the concentrations measured in the other phases to this point. For S. thermophilus LMD-9 Δ*comX* and E. faecalis JH2-2, fecal concentrations were compared within each phase. Comparisons were performed using the nonparametric Friedman test for repeated measures, followed by Dunn’s multiple-comparison test. The level of significance was set at a *P* value of <0.05. All calculations were performed with the GraphPad Prism software (version 7.03; La Jolla, CA, USA).
