# Supplementary material for: Transfer of the Integrative and Conjugative Element ICESt3 of Streptococcus thermophilus in Physiological Conditions Mimicking the Human Digestive Ecosystem
Source: Microbiol Spectr. 2023 Mar 30;11(3):e04667-22. doi: 10.1128/spectrum.04667-22 (PMC10269554; doi:10.1128/spectrum.04667-22)
Supplement: Supplemental file 1 — Supplemental material. Download spectrum.04667-22-s0001.pdf, PDF file, 0.10 MB [file spectrum.04667-22-s0001.pdf]

Table S1. Free donor and recipient strains released from beads (CFU) enumerated in cumulated ileal effluents and final jejuno-ileal content after 5-h in vitro milk digestion in the TIM-1 system. Median and interquartile range in brackets of three independent experiments. Abbreviations used: *S.*, *Streptococcus*; *E.*, *Enterococcus*. CFU: Colony-forming Unit.

	S. thermophilus LMG18311(ICE*St3*) x *E. faecalis* JH2-2(pMG36e)		*S. thermophilus* LMD-9(ICE*St3*) x *S. thermophilus* LMG18311(pMG36e)		*S. thermophilus* LMD-9(ICE*St3*) x *S. thermophilus* LMD-9 Δ*comX*		*S. thermophilus* LMD-9(ICE*St3*) x *E. faecalis* JH2-2(pMG36e)	
	Donor	Recipient	Donor	Recipient	Donor	Recipient	Donor	Recipient
Ileal effluent 0-5h	2.4×10^4 (2.8×10^4)	2.2×10^6 (8.0×10^5)	0 (0)	0 (0)	0 (0)	0 (0)	0 (2.2×10^3)	3.3×10^6 (2.8×10^6)
Final jejuno-ileal content	0 (0)	4.6×10^4 (8.8×10^4)	0 (0)	0 (0)	0 (0)	0 (0)	0 (1.3×10^3)	7.6×10^4 (5.7×10^5)

Table S2. Survival rates (%) of donor and recipient strains in beads after a 2 to 24h-incubation in the ARCOL system simulating healthy human colonic environment. Data of one experiment (n=1) per condition. Percentages are obtained by comparing the number of CFU per bead at each time of the experiment with the number of CFU of bacteria per bead at initial time. A percentage higher than 100% indicates a multiplication of the bacteria whereas a percentage lower than 100% indicates death of the bacteria. Abbreviations used: *S.*, *Streptococcus*; *E.*, *Enterococcus*.

Experiment in ARCOL	#1				#2			
Incubation time of beads	4h		24h		2h		5h	
Donor x Recipient	Donor	Recipient	Donor	Recipient	Donor	Recipient	Donor	Recipient
S. thermophilus LMG18311(ICE*St3*) x *E. faecalis* JH2-2(pMG36e)	6.1	55.3	0	2.6	8.6	79.5	1.1	89.7
S. thermophilus LMD-9(ICE*St3*) x *S. thermophilus* LMG18311(pMG36e)	8.4	3.4	4.8	0.6	114.9	52.4	38.7	7.8
S. thermophilus LMD-9(ICE*St3*) x *S. thermophilus* LMD-9 Δ*comX*	15.0	93.2	0.1	0.5	108.1	120.0	16.6	108.9
S. thermophilus LMD-9(ICE*St3*) x *E. faecalis* JH2-2(pMG36e)	43.3	112.4	0.02	3.5	37.9	63.6	5.3	141.6
